# Machine-learning-based risk stratification for probability of dying in patients with basal ganglia hemorrhage

**DOI:** 10.1038/s41598-022-25527-1

**Published:** 2022-12-05

**Authors:** Lili Guo, Nuoyangfan Lei, Mou Gao, Wenqiao Qiu, Yunsen He, Qijun Zhao, Ruxiang Xu

**Affiliations:** 1 Department of Neurosurgery, Sichuan Provincial People’s Hospital, University of Electronic Science and Technology of China, Chengdu, China; 2 Chinese Academy of Sciences Sichuan Translational Medicine Research Hospital, Chengdu, 610072 China; 3 College of Computer Science, Sichuan University, No.24 South Section 1, Yihuan Road, Chengdu, 610065 China; 4 State Key Laboratory of Fundamental Science on Synthetic Vision, College of Computer Science, Sichuan University, Chengdu, 610064 Sichuan China; 5 Department of Neurosurgery, Chinese PLA General Hospital, Beijing, 100853 China

**Keywords:** Biomedical engineering, Electrical and electronic engineering, Neuroscience, Diseases, Health care, Neurology, Risk factors, Mathematics and computing, Nanoscience and technology

## Abstract

To confirm whether machine learning algorithms (MLA) can achieve an effective risk stratification of dying within 7 days after basal ganglia hemorrhage (BGH). We collected patients with BGH admitted to Sichuan Provincial People’s Hospital between August 2005 and August 2021. We developed standard ML-supervised models and fusion models to assess the prognostic risk of patients with BGH and compared them with the classical logistic regression model. We also use the SHAP algorithm to provide clinical interpretability. 1383 patients with BGH were included and divided into the conservative treatment group (CTG) and surgical treatment group (STG). In CTG, the Stack model has the highest sensitivity (78.5%). In STG, Weight-Stack model achieves 58.6% sensitivity and 85.1% specificity, and XGBoost achieves 61.4% sensitivity and 82.4% specificity. The SHAP algorithm shows that the predicted preferred characteristics of the CTG are consciousness, hemorrhage volume, prehospital time, break into ventricles, brain herniation, intraoperative blood loss, and hsCRP were also added to the STG. XGBoost, Stack, and Weight-Stack models combined with easily available clinical data enable risk stratification of BGH patients with high performance. These ML classifiers could assist clinicians and families to identify risk states timely when emergency admission and offer medical care and nursing information.

## Introduction

Intracerebral hemorrhage (ICH) is a devastating neurosurgical emergency with significant morbidity and mortality, among which basal ganglia is the most common site, accounting for 50–70%^1^. Less than 50% of patients die within 30 days after hemorrhage, 30% of survivors have disabilities to varying degrees, and only 12–39% are capable of living independently 6 months after surgery^2,3^. At present, the main treatment options for BGH are conservative and surgical treatment. The selection of which treatment to be used urgently need to be done by the entire team ideally, while numerous factors include transient or permanent neurological deficits, the potential risk for surgery and the patient or their family’s wishes impact the final therapeutic option. Moreover, there is no unified standard treatment scheme for BGH.

Risk stratification is at the core of the medical practice, and providers need to risk stratify and identify patients at high risk. To date, typically, accurate decisions are time-consuming, while fast decisions are often inaccurate. Cutting time off the decision-making process ultimately means that life-saving and prognosis-improving interventions can get to the need much faster than has been seen historically.

Machine learning algorithms (MLA) based on big data are increasingly applied for the development of prediction models, disease diagnosis, and identification of risk factors^4–6^. Like other traditional clinical modules, neurologists have developed some traditional scoring systems according to the basic characteristics of patients with intracerebral hemorrhages, such as the ICH Score^7^, acute physiology and chronic health evaluation (APACHE) system^8^, and even the Glasgow Coma Scale (GCS) score^9^ to estimate hospital mortality. But the scoring results need to be evaluated by more experienced radiologists and/or neurologists, and as a consequence, they are affected by physicians’ clinical experience. Predictions based on data mining and MLA have continued to be focal points for over half a century. Various machine learning (ML) models have been applied to cerebrovascular disorders^10^, neurodegenerative disorders^11^, seizure^12^, cancer metastasis^13^, and COVID-19 prediction^14^. At present, a large number of studies have used MLA to construct and verify disease prediction models and proved that MLA plays a positive auxiliary role in clinical disease management and decision-making. Yet, there is a paucity of literature focused on stratifying prognosis in patients with BGH by examining the clinic characteristics using MLA.

The study of prognostic factors has important clinical guiding significance, and evaluating the risk of early death is the first step to deciding on therapeutic options and judging the prognosis. For the existing clinical decisions, our study aims to develop conservative treatment models and surgical treatment models to stratify the risk of dying within 7 days in patients with BGH, providing a promising early prediction method compared with other scoring systems in clinical practice.

## Methods

### Data source and clinical outcomes

#### Data source

**Figure 1:**
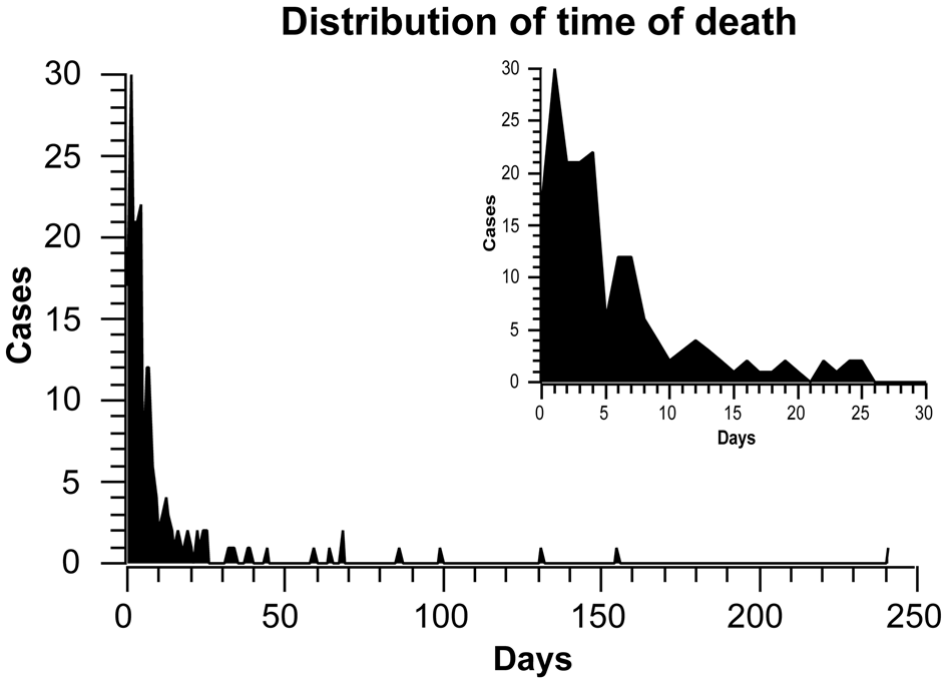
Distribution of time to death in patients with basal ganglia hemorrhage. The upper right corner shows deaths within 30 days of study subjects.

**Figure 2:**
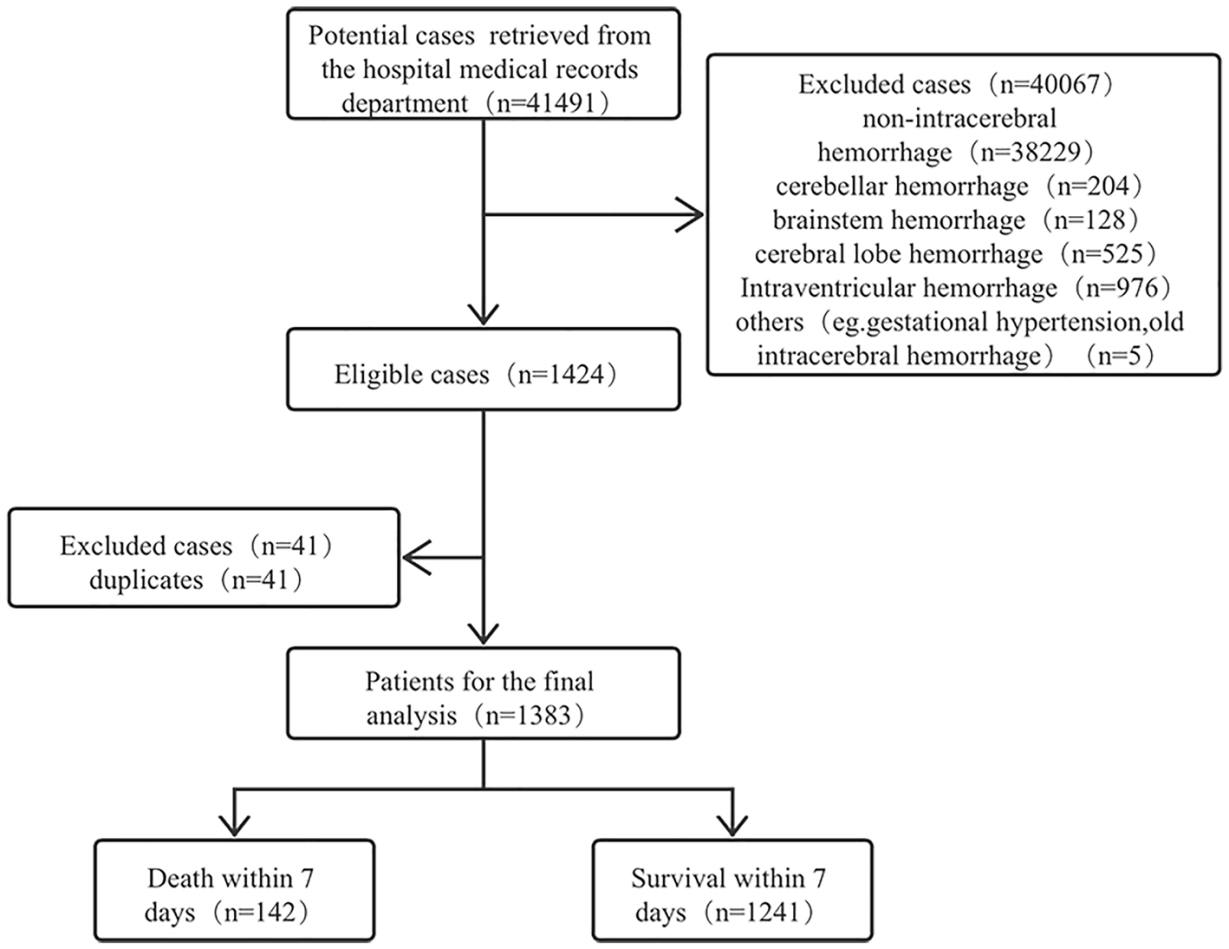
Flow diagram of study recruitment and exclusion.

1383 cases with BGH were first diagnosed by computerized tomography in the neurosurgery and emergency department of our hospital and with complete clinical data from 1 August, 2005 to 1 August 2021 were selected (Supplementary Table 1). The first issue to be considered in this study was how to select the time point of death to classify the high-risk group from the low-risk group. By observing the survival time of all patients who met the inclusion criteria, we found that the mortality rate decreased suddenly on the 5th and 7th days after BGH (Fig. 1). We then divide the period according to these two-time points and perform a two-sample independent *t*-test, and the results showed a cut-off at day 5 after BGH (*P* < 0.001, *t* = 5.7789) versus day 7 after BGH (*P* < 0.001, *t* = 6.4059). Therefore, we used whether the survival time after BGH was greater than seven days as a criterion to classify low risk versus high risk (Fig. 2). Also, patients were assigned to the conservative treatment group (CTG) and surgical treatment group (STG) based on whether surgery was selected. All processes of this study conformed to the ethical standards of the institutional and national medical ethics committees, as well as to the 1964 Declaration of Helsinki and similar ethical standards. This study was approved by the Medical Ethics Committee of Sichuan Provincial People's Hospital (approval number: 2022-154), and the committee waived the requirement for written informed consent because of the retrospective nature of this study.

#### Evaluation of clinical outcomes

The demographic data and clinical information were carefully gathered retrospectively from medical records, including gender, age, body type, prehospital time, smoking history, stroke history, complications (hypertension, hyperlipidemia, diabetes mellitus, epilepsy, brain edema, brain herniation, subarachnoid hemorrhage (SAH), lobar hemorrhage, fracture), pupil, hemorrhage breaking into ventricles (HBIV), hematoma volume measured by CT and laboratory examination indexes (hemoglobin, high-sensitivity C-reactive protein [hsCRP], consciousness at admission, blood pressure, pulse, and body temperature, intraoperative hematoma volume, bleeding volume, blood transfusion/fluid volume, operation time). Laboratory indexes are measured based on the data collected for the first time at admission; hematoma volume was calculated by applying the ABC/2 method, whereas A is the longest diameter (cm), B is the widest diameter (cm) and C represents the sum of the thickness of slides of hematoma in the CT scans^15^. The patients were dichotomized into two groups, the “high-risk” group with brain death declared within 7 days after admission and the “low-risk” group with survival longer than 7 days. Also, patients were assigned to CTG and STG based on whether surgical decisions (including decompressive craniectomy, external ventricular drainage, craniotomy evacuation of hematoma, and micro-invasive hematoma removal) were made. The relevant prognostic risk factors were analyzed.

Hypertension is defined as having a clear history of hypertension in the past or having multiple blood pressure measurements greater than 140/90 mmHg in this admission. According to the standard of the American Diabetes Association (2014), diabetes is diagnosed as follows: (1) fasting blood glucose is more than 7 mmol/L; (2) after oral glucose tolerance test, blood glucose is more than 11.1 mmol/L after 2 h; (3) patients with diabetes symptoms have random blood glucose equal to 11.1 mmol/L; (4) diabetes mellitus history or taking hypoglycemic drugs. Alanine aminotransferase > 50 IU/L is defined as abnormal liver function. Triglyceride > 1.7 mmol/L or total cholesterol > 5.2 mmol/L, with or without LDL cholesterol > 3.1 mmol/L is defined as hyperlipidemia. Smoking history was defined as patients revealing a history of smoking for > two pack-years and current smoking. Cardiovascular diseases include a history of myocardial blood deficiency, a history of myocardial infarction, arrhythmia (atrial fibrillation, ventricular fibrillation, bundle branch block above grade II, etc.), and heart failure. Renal insufficiency is defined as eGFR < 60 mL/min/1.73 m^2^ or serum creation clearance rate ≤ 104 mmol/L in the latest half-year. Infection includes pulmonary infection, urinary infection, HIV, HBV, and HCV.

### Model algorithms

#### Overview of the framework

**Figure 3:**
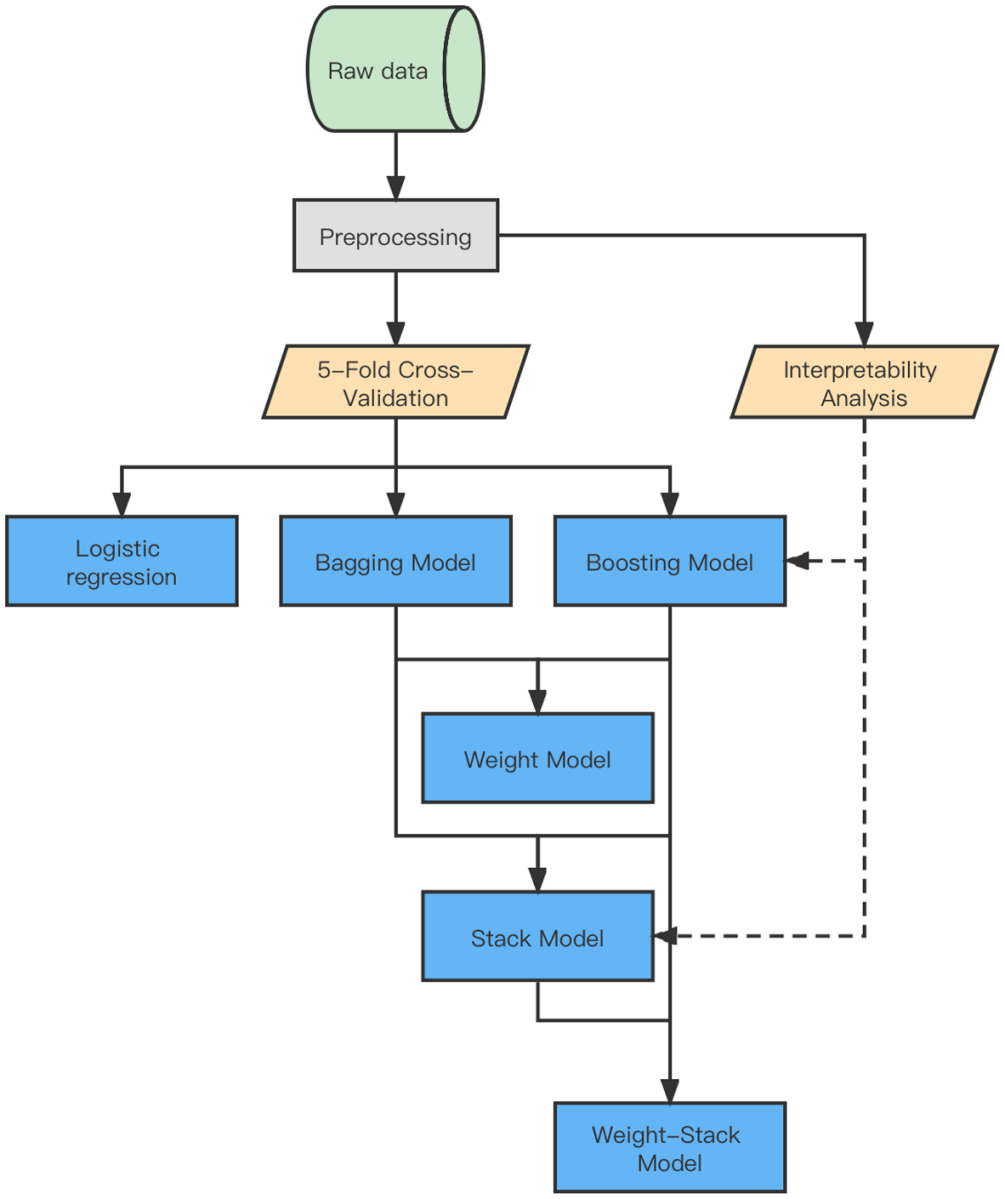
Overall workflow summarizing model algorithm.

We propose an analytical framework for the BGH risk stratification problem (Fig. 3). It includes three steps: data preprocessing, model construction, and interpretability analysis. Data cleaning, vector coding, and data resampling were the first to be applied to the raw data. We used the pre-processed balanced data as training input for model construction. The dataset was divided into training and testing sets in the ratio of 7:3. We built standard ML models using Boosting^16^ and Bagging^17^ methods, and fusion ML models integrated with standard ML models to improve the performance of risk stratification and provide effective clinical guidance. Finally, we chose the two models with better performance for the interpretability analysis of risk stratification.

#### Data preprocessing

We first performed data cleaning based on clinical a priori knowledge, removed conflicting or commonsense violating samples, and excluded some features irrelevant to the results and very small sample sizes. We also standardized coding on some variables: binary features such as gender, smoking, and brain herniation were coded 0–1, with 0 for negative and 1 for positive; among multiple categorical features, blood type was coded 1–5 (4 conventional blood types versus unchecked), and pupils were coded 1–6 according to whether they were equilibrated and normal/reduced/dilated, etc. In addition, prehospital time and operative time units were standardized to days or hours. In addition, we use the K-nearest neighbor (KNN) algorithm to fill some missing value features^18^. A total of 40 features were selected and the sample was classified as CTG or STG according to whether surgery was performed. finally, we built the model and implemented a binary classification to predict patient risk. After data preprocessing, we obtain a dataset of 1383 records, 743 and 640 records belonging to the CTG and STG, respectively.

### ML models

We chose traditional statistical methods: logistic regression (LR) for performance reference, three standard machine learning models based on Bagging or Boosting, Bagging: Random Forest (RF)^19^, Boosting: XGBoost^20^ and LightGBM^21^, and three fusion ML methods: Weight, Stack^22^, and Weight-Stack models for Electronic Health Records (EHR) data.

1. *Weight* The Averaging-like approach is used to assign different weights to the prediction results of multiple models to aggregate their prediction probabilities and improve the comprehensive performance. We need to take into account both the detection rate of high-risk patients and the misclassification rate, and finally, choose the F1 score as the weight of the model.
2. *Stack* A model layering approach is used. The prediction probability vectors based on each training and test set are first obtained using multiple lower-layer models, which are then integrated separately and input to the upper-layer model for retraining to obtain the final prediction labels. The upper-layer model in Stack takes the prediction probabilities as input instead of the original data and uses K-fold cross-validation internally to address possible overfitting problems.
3. *Weight-Stack* Combining the use of Weight and Stack methods, the better performing model is selected from the base models and weighted again with the Stack model using the F1 score as weight.

Weight and Stack use different approaches to integrate the advantages of heterogeneous models and improve predictive performance.

We used the following three points to avoid over-fitting situations: (1) the parallel generation of the prediction function in our chosen Bagging model can reduce the variance of the model prediction. (2) we perform cross-validation in the training set, giving the Bagging and Boosting models a restricted hyperparameter (number of base learners). (3) appropriate data cleaning to improve the volume and comprehensiveness of data distribution can effectively prevent overfitting. We use a combination of under-sampling and over-sampling to balance the training set data and perform data augmentation to get an accurate training model.

### Imbalanced data

In the dataset, the incidence of death within 7 days (high-risk) is low at 10.3%. This indicates that there is a category imbalance in our data, meaning an extremely significant difference in the sample size between the different categories^23^. By training on an unbalanced dataset, the model will focus more on most categories, leading to reduce the overall performance of the model^24^. To decrease the cost of misclassification, we must address the problem of misclassification of high-risk categories. We use the following methods to deal with imbalanced data: 1. add hyperparameters to provide higher class weights for small sample classes. 2. introduce the SMOTEEN algorithm based on the EE algorithm and the SMOTE algorithm, which uses a combination of oversampling and undersampling to solve the problem of lack of minority class samples and noise interference^25^. We load the SMOTEEN algorithm on the training set so that the model learns from balanced data and copes with the challenges posed by an imbalanced testing set. Ultimately, the training set samples rose from 520 to 737 in CTG and from 448 to 601 in STG. In addition, it was ensured that the ratio of high-risk to low-risk patients was approximately 1:1.

### Features and interpretability

ML is a "black box" in which clinical managers can only see the prediction results of a model, but not the origin of the results, making it difficult to be accepted by physicians in clinical practice. To address the problem of poor feature referencing and interpretability, we use the SHAP algorithm to interpret the risk stratification results and provide feature importance and correlation analysis for the whole or individual^26^. Avoiding the generalization problem of a certain model, we piggyback the SHAP algorithm on a standard ML model together with a fusion model to provide interpretability.

### Statistical analysis

We use receiver operating characteristic curves (ROC) and precision-recall curves (PR) to evaluate the performance of each model. The ROC curves do not reflect true model binary classification performance when the data are unbalanced, so we combine them with the results of the PR curves analysis to analyze the results more comprehensively for a small number of categories of samples^27^. The area under the ROC curves (AUC/ROC) is presented as a measure of the ROC curves’ performance, as well as the area under the PR curves (AUC/PR) using the average precision score as an approximation of the PR curve’s performance measure^28^. We also report the sensitivity (Recall), specificity, accuracy, precision (PPV), negative predictive value (NPV), and F1 score for each model. All reports were obtained by using the recommended hyperparameters from the fivefold cross-validation and by repeating the experiments. Descriptive statistics were performed on categorical and numerical variables. Numerical variables were presented with means (standard deviation [SD]), while numbers (%) were used for categorical variables. Two groups were compared by the Wilcoxon rank sum test for paired data in non-normal distribution, and by a *t*-test of paired samples in a normal distribution. The significance level was set at *P* < 0.05.

## Results

### Patients characteristics

Overall 1383 patients with BGH in 41,491 patients fitting the inclusion criteria were enrolled in the study. The baseline characteristics of the data were shown in Supplementary Table 1. Among them, the overall mortality rate was 16.41% (195/1383), (CTG, 114/743[15.34%]; STG, 81/640, [12.66%]); the median age was 57 years (interquartile range, 48–66), and the majority of patients were male (913/1383, 66%). The median length of stay was 13 days.

### Model performance

Table 1 shows the AUC/ROC and AUC/PR for all models in CTG and STG. In parameter optimization, to ensure generalization, all models were adjusted for the maximum number of iterations hyperparameter by the grid search method only (Supplementary Data 1). In the testing result, regarding the risk stratification of STG, the risk stratification performance of the models (AUC/ROC: 0.696–0.820, AUC/PR: 0.231–0.361) was generally lower than that of CTG (AUC/ROC: 0.906–0.925, AUC/PR: 0.576–0.662) (Table 1). The commonly used AUC/ROC model performance evaluation methods have a low ability to capture low accuracy problems in unbalanced binary classification. This can lead to the actual performance of the model being exaggerated and it is difficult to reflect the difference^29^. We need to pay attention to both the AUC/PR curves’ performanceandr other metrics under the optimal classification threshold selected by the model to reflect the model performance comprehensively.

**Table 1:** AUC/ROC and AUC/PR in conservative versus surgical treatment groups (from s.d.).

	Random forest	XGBoost	LightGBM	LR	Weight	Stack	Weight-stack
**Training**							
Conservative treatment							
AUC/ROC	0.990 (0.985–0.994)	0.969 (0.964–0.975)	0.974 (0.970–0.977)	0.937 (0.928–0.945)	0.989 (0.984–0.993)	0.990 (0.985–0.994)	0.984 (0.979–0.988)
AUC/PR	0.917 (0.888–0.946)	0.732 (0.684–0.780)	0.754 (0.697–0.810)	0.701 (0.673–0.729)	0.908 (0.878–0.938)	0.915 (0.877–0.952)	0.869 (0.836–0.901)
Surgical treatment							
AUC/ROC	0.981 (0.975–0.987)	0.966 (0.957–0.976)	0.977 (0.970–0.984)	0.765 (0.755–0.775)	0.980 (0.973–0.987)	0.981 (0.974–0.987)	0.977 (0.969–0.985)
AUC/PR	0.893 (0.883–0.903)	0.696 (0.647–0.745)	0.782 (0.751–0.813)	0.265 (0.223–0.306)	0.885 (0.874–0.896)	0.876 (0.864–0.888)	0.838 (0.812–0.864)
**Testing**							
Conservative treatment							
AUC/ROC	0.925 (0.909–0.941)	0.906 (0.886–0.925)	0.913 (0.899–0.926)	0.923 (0.899–0.948)	0.921 (0.904–0.938)	0.923 (0.905–0.941)	0.918 (0.900–0.936)
AUC/PR	0.601 (0.510–0.691)	0.585 (0.502–0.669)	0.576 (0.514–0.639)	0.662 (0.605–0.720)	0.602 (0.526–0.678)	0.621 (0.529–0.713)	0.609 (0.525–0.693)
Surgical treatment							
AUC/ROC	0.820 (0.802–0.838)	0.798 (0.777–0.819)	0.787 (0.764–0.809)	0.696 (0.654–0.737)	0.811 (0.784–0.837)	0.820 (0.793–0.846)	0.805 (0.783–0.828)
AUC/PR	0.334 (0.270–0.398)	0.341 (0.268–0.413)	0.341 (0.244–0.439)	0.231 (0.186–0.276)	0.348 (0.271–0.425)	0.361 (0.276–0.447)	0.361 (0.296–0.427)

Table 2 gives the model performance for CTG and STG risk stratification at a 95% confidence interval (CI). In ensuring the overall accuracy of the model, we intend to shed more light on the high-risk category samples, especially the misclassified high-risk samples. In the ability to detect high-risk patients, the Stack model in CTG had the highest sensitivity rate of 78.5%. And in STG, even though we need to focus on high-risk class samples and LR has a high sensitivity (69.1%) compared to XGBoost (61.4%), its poor performance in specificity (48.4%) and accuracy (50%) compared to XGBoost (specificity: 82.4%, accuracy: 80.5%). Based on the above report, we chose XGBoost in standard models with Stack model in fusion models loaded with SHAP algorithm for risk-stratified binary classification prediction for interpretability analysis.

**Table 2:** Assessment metrics for the conservative versus surgical treatment groups on the testing set, including: F1, sensitivity (recall), precision (PPV), specificity, NPV and accuracy (from s.d.).

	Fl	Sensitivity (recall)	Precision (PPV)	Specificity	NPV	Accuracy
**Conservative treatment**						
Random forest	0.559 (0.509–0.608)	0.691 (0.619–0.764)	0.472 (0.416–0.529)	0.902 (0.882–0.923)	0.959 (0.948–0.970)	0.879 (0.864–0.895)
XGBoost	0.548 (0.517–0.579)	0.744 (0.667–0.820)	0.438 (0.395–0.481)	0.880 (0.859–0.902)	0.965 (0.952–0.977)	0.865 (0.853–0.877)
LightGBM	0.546 (0.505–0.588)	0.735 (0.631–0.839)	0.442 (0.390–0.494)	0.882 (0.846–0.917)	0.964 (0.948–0.980)	0.865 (0.841–0.890)
LR	0.546 (0.470–0.622)	0.462 (0.382–0.542)	0.678 (0.568–0.788)	0.972 (0.960–0.983)	0.935 (0.919–0.950)	0.914 (0.895–0.934)
Weight	0.556 (0.523–0.590)	0.736 (0.650–0.821)	0.454 (0.403–0.505)	0.887 (0.858–0.916)	0.964 (0.951–0.977)	0.870 (0.852–0.889)
Stack	0.572 (0.528–0.617)	0.785 (0.707–0.863)	0.457 (0.396–0.517)	0.880 (0.848–0.913)	0.970 (0.957–0.983)	0.869 (0.845–0.893)
Weight-stack	0.568 (0.535–0.602)	0.742 (0.648–0.837)	0.467 (0.416–0.518)	0.892 (0.865–0.919)	0.965 (0.951–0.979)	0.876 (0.860–0.891)
**Surgical treatment**						
Random forest	0.352 (0.322–0.382)	0.537 (0.479–0.595)	0.267 (0.226–0.307)	0.862 (0.831–0.892)	0.952 (0.940–0.964)	0.834 (0.807–0.860)
XGBoost	0.347 (0.303–0.392)	0.614 (0.536–0.692)	0.245 (0.200–0.289)	0.824 (0.795–0.852)	0.957 (0.944–0.971)	0.805 (0.777–0.834)
LightGBM	0.353 (0.288–0.419)	0.550 (0.482–0.619)	0.264 (0.203–0.325)	0.850 (0.801–0.899)	0.952 (0.939–0.965)	0.824 (0.775–0.873)
LR	0.189 (0.165–0.213)	0.691 (0.593–0.789)	0.111 (0.093–0.128)	0.484 (0.398–0.570)	0.942 (0.921–0.964)	0.500 (0.425–0.576)
Weight	0.353 (0.308–0.398)	0.556 (0.532–0.580)	0.260 (0.213–0.307)	0.851 (0.822–0.880)	0.954 (0.945–0.962)	0.826 (0.797–0.856)
Stack	0.377 (0.324–0.430)	0.562 (0.506–0.619)	0.287 (0.232–0.342)	0.867 (0.834–0.901)	0.954 (0.943–0.966)	0.841 (0.807–0.875)
Weight-stack	0.367 (0.326–0.408)	0.586 (0.536–0.636)	0.269 (0.226–0.311)	0.851 (0.824–0.878)	0.956 (0.944–0.968)	0.828 (0.799–0.858)

**Figure 4:**
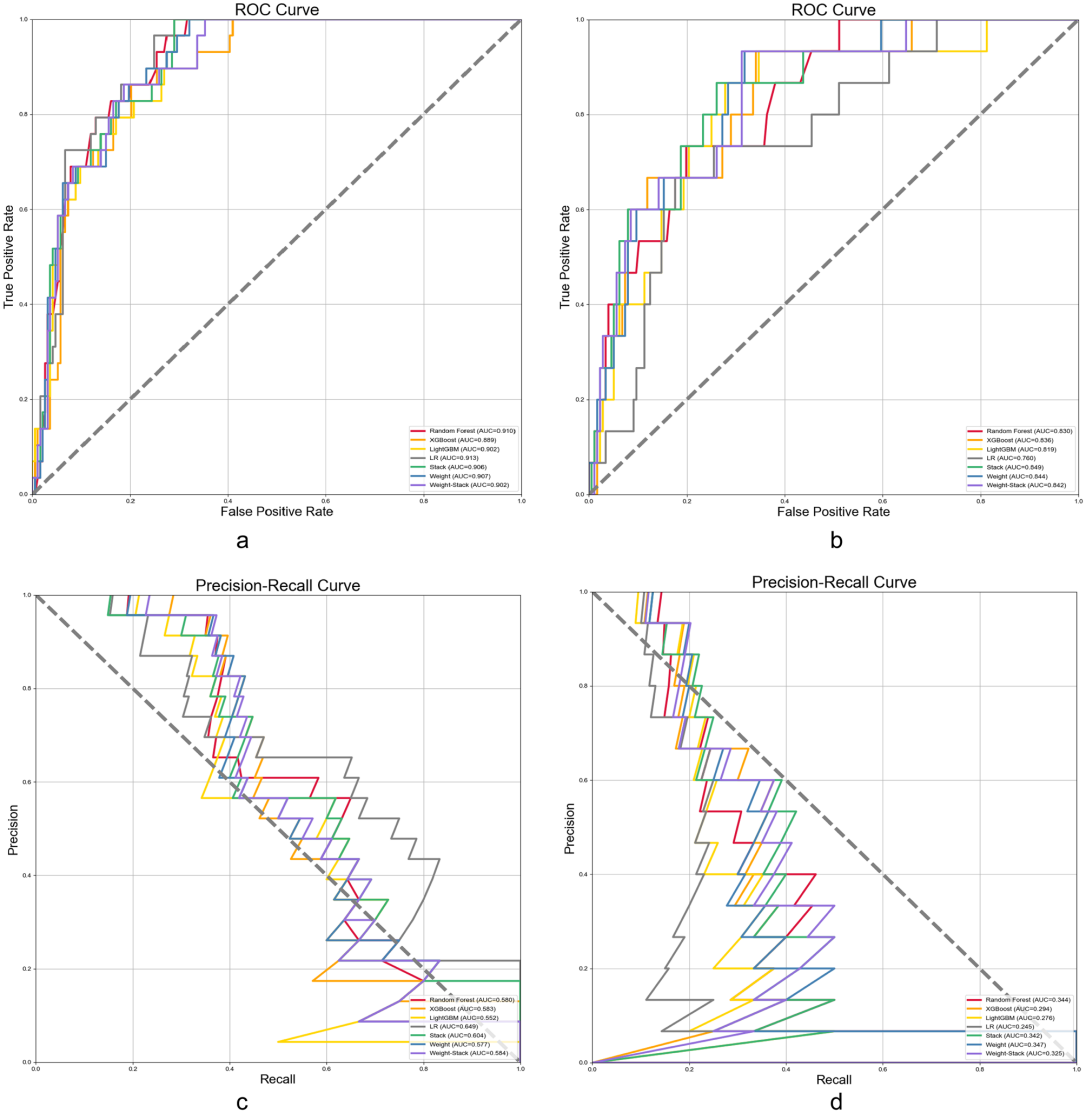
Receiver operating characteristic curves versus precision-recall curves for the conservative treatment group and the surgical treatment group. (**a**) Receiver operating characteristic curves for all models in the conservative treatment group. (**b**) Receiver operating characteristic curves for all models in the surgical treatment group. (**c**) Precision-recall curves for all models in the conservative treatment group. (**d**) Precision-recall curves for all models in the surgical treatment group.

Figure 4 reports the ROC vs. PR curves based on CTG vs. STG in the testing (see Supplementary Fig. 1 for training). As shown in Fig. 4. First, the ROC and PR curves of CTG perform significantly better than STG; in the ROC curve, all models show similar trends, but in the PR curve, the different models show more trend differences. In addition, we report the calibration curves of CTG and STG in separate training and testing (Supplementary Fig. 2).

### Interpretability analysis

#### Group-level analysis

**Figure 5:**
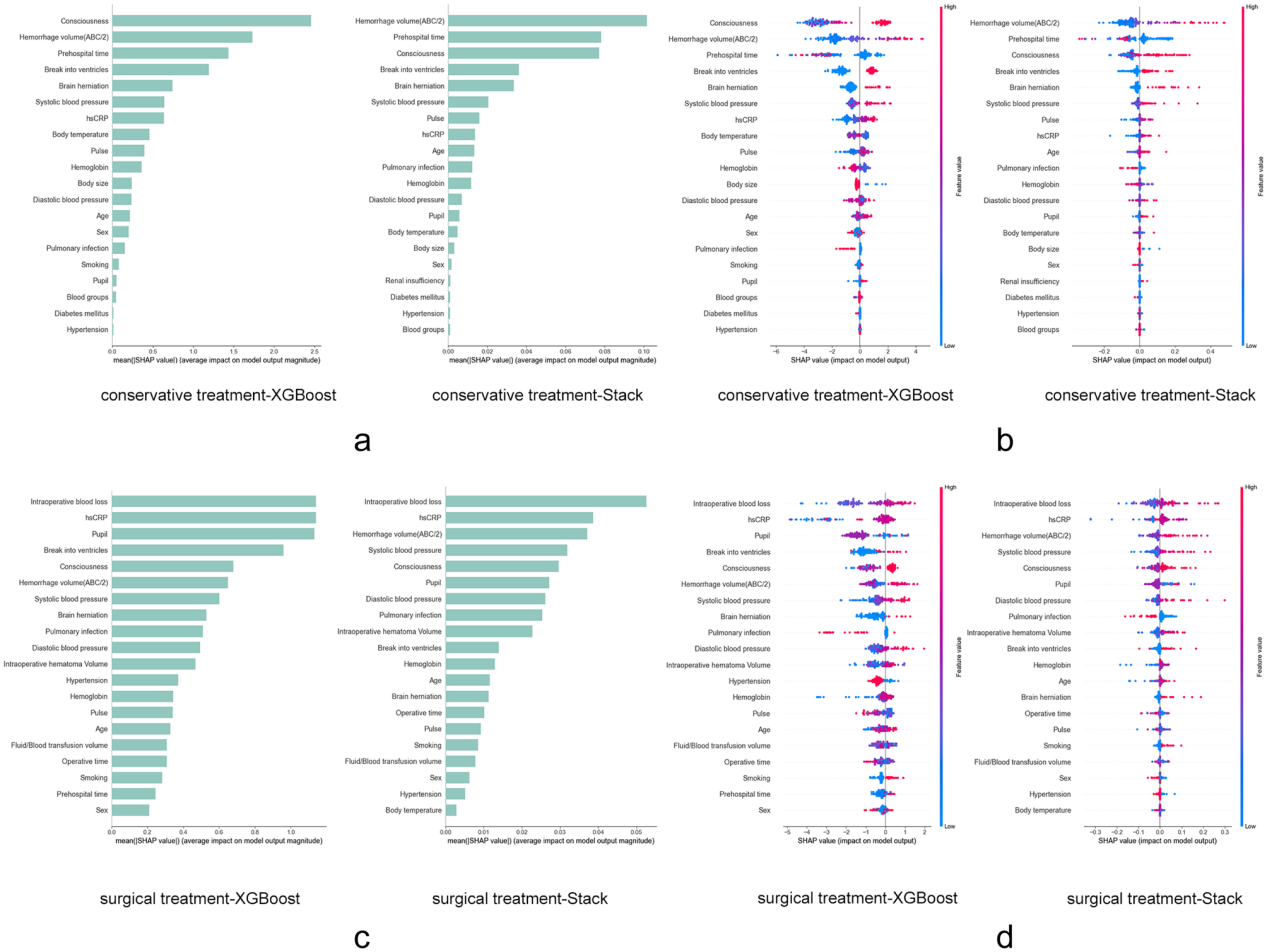
Overall SHAP interpretation of XGBoost and Stack model risk stratification. XGBoost uses the tree model interpretation method and the Stack model uses the Kernel interpretation method. (**a**, **c**): Ranking of all features in order of importance and showing the top 20 features. (**b**, **d**): Distribution showing the correlation of features with risk stratification results. Each point is a sample, red dots represent a high value of features, blue dots represent a low value of features, a negative SHAP value indicates a decrease in the probability of high risk and a positive one indicates an increase in the probability of high risk.

Figure 5a and b provide a detailed view of the CTG risk stratification features reported with the XGBoost and Stack model. At the population level, both models identified poor patient awareness on admission, high hematoma volume by the ABC/2 method, a short time from onset to admission, hematoma breaking into the ventricle, and the presence of brain herniation as the most influential features determining high-risk stratification (Fig. 5a and b). Characteristics such as age, hsCPR, pulse, and blood pressure were relatively weak factors influencing risk stratification (Fig. 5a).

For the group of patients who underwent surgical treatment, Fig. 5c and d show partially different perspectives. First, the XGBoost and Stack model consider increased intraoperative blood loss, high level of hsCRP, poor consciousness, and high blood pressure as the most influential features that predict increased risk, while intraoperative transfusion volume, brain herniation, and hematoma volume obtained by the ABC/2 method also play an important role (Fig. 5d). Notably, there is a tendency for low-risk stratification in the pulmonary infection group (Fig. 5b,d), and this would depend on the combination with other characteristics.

#### Individual-level analysis

**Figure 6:**
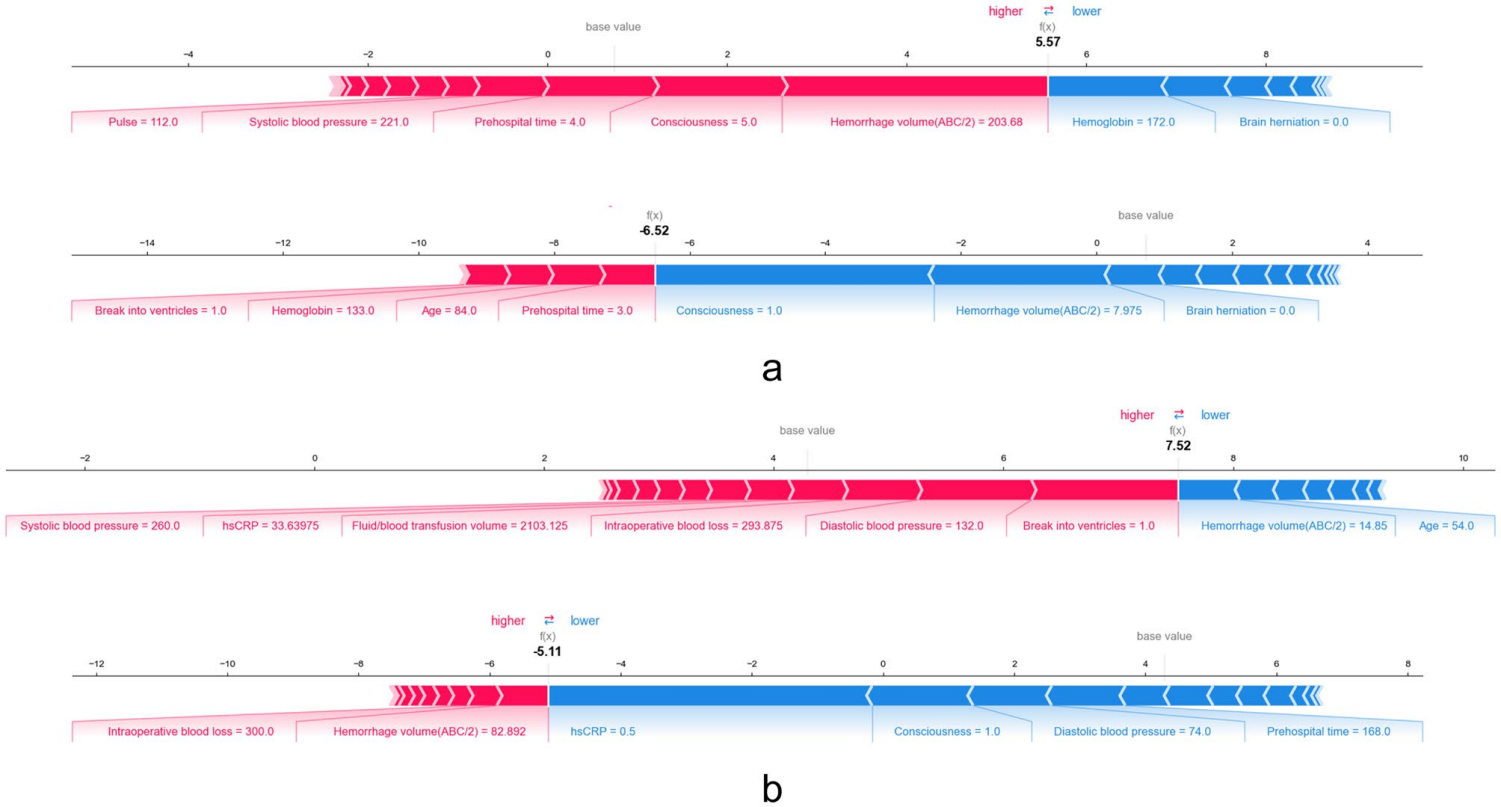
SHAP force plots based on individual patient prediction scores. (**a**) Low- and high-risk instances in the CTG. (**b**) Low- and high-risk instances in the STG. the base value is the mean Shap values predicted by the model, and the red and blue directed bars indicate the risk and safety features, respectively; the length of the bars represents the importance of the feature, which together drive the prediction from the base to the final value. The Shap values shown in the graph are the log odds of the true predicted probability.

On a holistic basis, we can also provide explanations for risk stratification at the individual level. For example, in the high-risk example of the CTG, enlarged hematoma volume and a coma level of consciousness on admission increased the predicted risk significantly, although hemoglobin at normal levels and without brain herniation could not stop the trend (Fig. 6a). In contrast, in the high-risk instances in STG, hematoma break into the ventricles, high blood pressure, and escalated intraoperative blood loss could increase the dying within 7 days risk (Fig. 6b). This allows for a clinical interpretation of the risk prediction for individual patients.

## Discussion

In this study, we aim to test whether the application of ML technology can improve the risk stratification performance of the prognosis (dying within 7 days) of BGH. To the best of our knowledge, there has been no attempt to predict, stratify, and interpret the risk of dying in patients with BGH using ML approaches based on more-than-ten-year clinical data. First, we collected a set of clinical samples of emergency admission related to patient prognosis derived from the Hospital Information System (HIS). A total of 40 features were selected from the original data based on clinical experience and correlation analysis, and the sample was divided into CTG and STG. These features are then fed into six different models, hyperparameters are tuned in the training set using the grid search method based on fivefold cross-validation. Training and testing performance were reported, including ROC, PR, calibration, AUC/ROC, AUC/PR, and statistical metrics. Finally, the SHAP algorithm provides interpretability for risk stratification of the model, including feature analysis from the whole to individual instances. Based on the model performance, we found that:

1. Standard and fusion ML models generally have higher risk stratification performance compared with the traditional analysis method of LR in statistics.
2. In terms of CTG sensitivity, the Stack model 0.785 (0.707–0.863) performed best. XGBoost was 0.744 (0.667–0.820) (*P* < 0.001) and Weight-Stack model 0.742 (0.648–0.837) (*p* < 0.05). In addition, the Stack model had advantages in the F1 score and NPV. In conclusion, the Stack model performed best in CTG.
3. In STG, excluding LR with poor classification ability (F1 score: 18.9%, accuracy: 50%), the sensitivity of XGBoost was 0.614 (0.536–0.692), which was better than that of Stack model (0.562 [0.506–0.619], *P* < 0.05). However, it was not significantly different from the Weight-stack model (0.586 [0.536–0.636], *P* = 0.189). Combining the other performances of the Weight-Stack model, it can be concluded that XGBoost and Weight-Stack performed best in STG.

However, considering the practical needs, clinical managers can also choose from these models on their own, depending on the situation.

This mainly lies in three reasons: First, the technique of Bagging and Boosting methods to assemble weak classifiers into strong classifiers plays an important role in the standard ML selected for our study. Second, ML models have an advantage when dealing with large amounts of data (more inclined to complex sample distributions), and the ML model we use performs better than LR, which, due to its simple form (linear-like model), has difficulty fitting the true distribution of complex samples. Third, considering the differences in training and prediction principles of different models, the fusion model uses the prediction probabilities of multiple ML standard models as input, combining the advantages of each model to achieve improved model performance.

Fusion models derived from multiple standard ML models paired with interpretable analysis algorithms are indeed good candidates to guide clinical administrators in the early individualized risk stratification of BGH. At present, the main treatment options for BGH are conservative and surgical treatment. Research shows that secondary clinical neurological deterioration generally occurs about 12 h after the onset of intracerebral hemorrhage, and cerebral ischemia can occur about 24 h after the formation of hematoma, which may aggravate the degree of motor impairment in patients with intracerebral hemorrhage. As a consequence of a reduction in hematoma volume, the intracranial pressure eventually decreases, and blood perfusion of brain tissue returns^30^. Furthermore, invasive hematoma removal may prevent secondary neurotoxicity and edema caused by thrombin and hemoglobin degradation products^31,32^. Thus, theoretically, hematoma evacuation quickly can be beneficial. The prognosis of patients with intracerebral hemorrhage is closely related to the location, volume, duration, and complications of intracerebral hemorrhage. Based on the ML classifier with the SHAP algorithm, we predict the 7-day outcome of patients by entering features that are quickly available at admission when conservative treatment is chosen; if surgical treatment is chosen based on guidelines, physician experience, or the patient's family's wishes, indicators that are easily available at admission and/or before surgery and some intraoperative features can together predict the 7-day postoperative outcome.

In conclusion, the ML classifier combining easily available routine clinical measurements with intraoperative information may create a new prognostic risk assessment strategy for BGH to identify high-risk patients undergoing surgery/conservative treatment. ML classifier will help clinical managers to pre-select high-risk patients and take necessary measures or monitoring to prolong their lives.

Our study shows that compared with the conservative treatment model, the intraoperative characteristic variables including inpatients have a poor ability to stratify the prognosis, which might imply that the relationship between intraoperative eigenvalues and prognosis was weak, so the next work is to collect as many postoperative characteristics and data as possible and screen out more influential prognostic factors, such as swelling shown by postoperative MRI image and pro-brain natriuretic peptide (pro-BNP)^33,34^.

Our study suggests that consciousness, hemorrhage volume (ABC/2), prehospital time, and break into the ventricles were the most important features in CTG. This is well understood. Generally, the larger the hematoma volume, the more obvious the activation of nerve compression and pathological process, so that emergency hospitalized patients are often accompanied by serious disorders of consciousness. The reaction time of the family is also shorter and more timely, which also explains that many risk stratification models focus on hematoma volume and level of consciousness, through E. Berkeveld found there was no association between prehospital time and mortality^35^. Initial hematoma volume is the strongest predictor of 30-day mortality^36^, and it is a threat to pre- and postoperative mortality rates^37,38^. Some scholars aim at the impact of different specific locations of hematoma on prognosis. Their results are consistent with this study. In this study, 57.97% of cases broke into the ventricle, and some studies similarly found that more than 60%^39^ of BGH would break into the ventricle. The reason for the small difference is that this study did not limit the volume of bleeding in patients with BGH diagnosed by CT. Different from other studies, this study did not find that pulmonary infection is very related to the prognosis of BGH. The reasons maybe are as follows: 1. for acute, critical, and severe cases, doctors tend to pay attention to factors threatening life, such as increased intracranial pressure caused by ICH, and easy to ignore whether it is complicated with pulmonary infection, resulting in unrecorded cases. 2. even if a vicious circle is formed between lung infection and poor prognosis in BGH, this factor that should have existed cannot be displayed when the patient dies rapidly. STG showed that intraoperative bleeding and hsCRP were the strongest predictors. The average operative time was 3.40 h, the average blood loss was 255.93 cc, the average blood transfusion and infusion volume was 2145.86 ml, and the average hsCRP was 33.55 mg/L (> 10 mg/L, 82.67% [529/640]). Therefore, minimally invasive puncture treatment can decrease the fatality rate to 33.3% (7/21)^40^. The hsCRP is an indication of inflammation, a prospective, observational study from Huangfu XQ found statistically that high a level of hsCRP appeared to be an independent predictor for 90-day death, overall survival, and poor outcome in acute primary BGH^41^.

## Limitations

First, our single-center retrospective study cannot cover all the potential characteristics associated with survival outcomes during admission. 1. Since patients were enrolled within a time frame of more than 10 years, we cannot rule out that specific changes in treatment philosophy or technology may affect the results; 2. errors from patients declining therapy or shortening their course of therapy and data records also affect their accuracy; and 3. although the performance of the algorithm has good results, the sample size is relatively small and comes from single-center data. The research with larger samples may obtain higher prediction ability. Therefore, our results may not be directly applicable to other centers or countries.

Second, the exact causal relationship and causes between these predictive variables and 7-day survival outcomes could not be determined. (1) This is an unavoidable disadvantage of cross-sectional research compared with prospective research, and (2) Our model cannot replace clinical judgment and result in risk stratification based on human long-term experience, and may still be better than the best algorithm. Therefore, the application of prognosis stratification in individual patients should be treated with caution.

Third, any model is bound to have some limitations. (1) The sample size for high prognostic risk is relatively small, accounting for only 10.2% of the total sample. Even though we use a data-balancing correlation algorithm, we could not fully improve the model's low attention to high-risk samples, which affected the accuracy of the model; (2) in the STG, fewer intraoperative indicators are included due to the limitation of the surgical record file in the database, which may have contributed to the larger difference in the risk stratification performance of the model; (3) models requires external validation to verify applicability at the clinical level.

## Supplementary Information

Dataset S1.

Dataset S2.

Supplementary Figure 1.

Supplementary Figure 2.

Supplementary Table 1.

## Data Availability

The hyperparametric design of the model can be found in Supplementary Data 1. The code for the dataset is available in Supplementary Data 2. In addition, Prof Ruxiang Xu can be contacted (rxiangxu@163.com) regarding the availability of data and materials.

## Abbreviations

ICH: Intracerebral hemorrhage
BGH: Basal ganglia hemorrhage
MLA: Machine learning algorithms
APACHE: Acute physiology and chronic health evaluation
GCS: Glasgow Coma Scale
SAH: Subarachnoid hemorrhage
HBV: Hemorrhage breaking into ventricles
hsCRP: High-sensitivity C-reactive protein
CTG: Conservative treatment group
STG: Surgical treatment group
LR: Logistic regression
RF: Random forest
