# Supplementary material for: Machine-learning-based risk stratification for probability of dying in patients with basal ganglia hemorrhage
Source: Sci Rep. 2022 Dec 5;12:21035. doi: 10.1038/s41598-022-25527-1 (PMC9722697; doi:10.1038/s41598-022-25527-1)
Supplement: Supplementary file 1 — Dataset S1. [file 41598_2022_25527_MOESM1_ESM.docx]

Supplementary Data 1. Hyperparameter usage for each model

Random Forest: n_estimators = 140

XGBoost: n_estimators = 160, scale_pos_weight = 5

LightGBM: n_estimators = 100

LR: N/A

Weight model: N/A

Stack model: meta_classifier = LogisticRegression(), cv = 5, use_probas = True

Weight-Stack model: meta_classifier = LogisticRegression(), cv = 5, use_probas = True
