# Supplementary material for: Machine-learning-based risk stratification for probability of dying in patients with basal ganglia hemorrhage
Source: Sci Rep. 2022 Dec 5;12:21035. doi: 10.1038/s41598-022-25527-1 (PMC9722697; doi:10.1038/s41598-022-25527-1)
Supplement: Supplementary file 2 — Dataset S2. [file 41598_2022_25527_MOESM2_ESM.pdf]

Supplementary Data 2. Code

```
import pandas as pd
import sklearn
import matplotlib.pyplot as plt
import xgboost as xgb
import lightgbm as gbm
import numpy as np
from sklearn import model_selection
from sklearn.metrics import
roc_curve,auc,roc_auc_score,average_precision_score,precision_recall_curve
from sklearn.model_selection import train_test_split
from sklearn import metrics
import matplotlib.pyplot as plt
plt.rc('font',family='Arial')
from sklearn.model_selection import GridSearchCV
import seaborn as sb
from  sklearn.ensemble import RandomForestClassifier
from imblearn.combine import SMOTEENN
from sklearn.linear_model import LogisticRegression
from mlxtend.classifier import StackingCVClassifier
import shap
from sklearn.calibration import calibration_curve


def loadDataset(filePath):
    df = pd.read_csv(filepath_or_buffer=filePath)
    return df


def featureSet(data):
    """ Read the features and labels in the file according to the number of features """
    data_num = len(data)
    XList = []
    yList = []
    for row in range(0, data_num):
        tmp_list = []
        for number in range(0,39):
            tmp_list.append(data.iloc[row][number])
        XList.append(tmp_list)
    for row in range(0, data_num):
        temp_list = []
```

```
        temp_list.append(data.iloc[row][39])
        yList.append(temp_list)
        """ Return 39 features and 1 label """
    return XList, yList


def loadTestData(filePath):
    """ Read the features and labels in the file according to the number of features """
    data = pd.read_csv(filepath_or_buffer=filePath)
    data_num = len(data)
    XList = []
    yList = []
    for row in range(0, data_num):
        tmp_list = []
        for number in range(0, 39):
            tmp_list.append(data.iloc[row][number])
        XList.append(tmp_list)
    for row in range(0, data_num):
        temp_list = []
        temp_list.append(data.iloc[row][39])
        yList.append(temp_list)
        """ Return 39 features and 1 label """
    return XList,yList


def traintest_XGB(X_train, y_train, X_test, y_test, X_train_raw, y_train_raw):
    """    XGBoost module    """
     #    model    =    xgb.XGBClassifier(objective="binary:logistic",    learning_rate=0.3,
n_estimators=220, max_depth=9, min_child_weight=1, subsample=0.7,
     #                        colsample_bytree=0.8, gamma=0.1, reg_alpha=3,
reg_lambda=2,scale_pos_weight=8)
    """ can use scale_pos_weight or other hyperparameters """
    model = xgb.XGBClassifier(n_estimators = 160, scale_pos_weight = 5)
    X_train = np.array(X_train)
    X_test = np.array(X_test)
    model.fit(X_train, y_train)
    """    SHAP for XGBoost    """
    explainer = shap.TreeExplainer(model)
    shap_values = explainer.shap_values(X_test)
    """ feature_names : Feature names array in dataset """
    """ Group level analysis chart of SHAP """
    shap.summary_plot(shap_values,                                X_test,
feature_names=['Feature_1','Feature_2','Feature_3'])
    shap.summary_plot(shap_values,                                X_test,
```

```
plot_type="bar",color='#92C7BE',feature_names=['Feature_1','Feature_2','Feature_3'])
    """ Individual level analysis chart of SHAP """
    Y_shap = np.array(y_test)
    for number in range(0,100):
        """ Label is 0 (negative) """
        if (Y_shap[number] == 0.0):
            shap.force_plot(explainer.expected_value, shap_values[number, :],
X_test[number, :], feature_names=[
                'Feature_1','Feature_2','Feature_3'], matplotlib=True, show=False)
    for number in range(0, 100):
        """ Label is 1 (positive) """
        if (Y_shap[number] == 1.0):
            shap.force_plot(explainer.expected_value, shap_values[number, :],
X_test[number, :], feature_names=[
                'Feature_1','Feature_2','Feature_3'], matplotlib=True, show=False)
            """ Save path """
            plt.savefig("Save_path"+str(number)+".png")

    """ XGBoost training results on the original training set """
    train_XGB_pre = model.predict(X_train_raw)
    train_XGB_proba = model.predict_proba(X_train_raw)
    y_train_predprob.append(train_XGB_proba[:, 1])

    """ XGBoost testing results on the testing set """
    test_XGB_pre = model.predict(X_test)
    test_XGB_proba = model.predict_proba(X_test)
    y_test_predprob.append(test_XGB_proba[:, 1])

    """ XGB training metrics """
    print('The XGBoost accuracy of the Train is:', metrics.accuracy_score(y_train_raw,
train_XGB_pre))
    print('The XGBoost F1 of the Train is:', metrics.f1_score(y_train_raw, train_XGB_pre))
    confusion_matrix_result = metrics.confusion_matrix(y_train_raw, train_XGB_pre)
    print('The XGBoost Train confusion matrix result:\n', confusion_matrix_result)

    """ XGB testing metrics """
    print('The XGBoost accuracy of the Test is:', metrics.accuracy_score(y_test, test_XGB_pre))
    print('The XGBoost F1 of the Test is:', metrics.f1_score(y_test, test_XGB_pre))
    confusion_matrix_result = metrics.confusion_matrix( y_test,test_XGB_pre)
    print('The XGBoost Test confusion matrix result:\n', confusion_matrix_result)
    plt.figure(figsize=(8, 6))
    sb.heatmap(confusion_matrix_result, annot=True, cmap='Blues',fmt='g')
    plt.xlabel('Predicted labels')
    plt.ylabel('True labels')
```

```
    plt.show()
    """ Return F1 score and prediction probability as the input of Weight """
    return metrics.f1_score(y_test, test_XGB_pre), test_XGB_proba,
metrics.f1_score(y_train_raw, train_XGB_pre), train_XGB_proba


def traintest_LGB(X_train, y_train, X_test, y_test, X_train_raw, y_train_raw):
    """   LightGBM module   """
    # clf=gbm.LGBMClassifier(boosting_type='gbdt', objective='binary',
    #                        colsample_bytree=0.5, learning_rate=0.04,
    #                        min_child_samples=7, min_child_weight=0.001,
    #                        n_estimators=500,  num_leaves=35,
    #                            random_state=None, reg_alpha=0.0,
reg_lambda=0.0, max_depth=7,
    #                        subsample=0.01,)
    """ can use  other hyperparameters """
    clf = gbm.LGBMClassifier(n_estimators=100)
    clf.fit(X_train,y_train)
    test_LGB_pre = clf.predict(X_test)
    test_LGB_proba = clf.predict_proba(X_test)

    """ LightGBM training results on the original training set """
    train_LGB_pre = clf.predict(X_train_raw)
    train_LGB_proba = clf.predict_proba(X_train_raw)
    y_train_predprob.append(train_LGB_proba[:, 1])
    confusion_matrix_result = metrics.confusion_matrix(y_train_raw, train_LGB_pre)
    print('The LightGBM accuracy of the Train is:', metrics.accuracy_score(y_train_raw,
train_LGB_pre))
    print('The LightGBM F1 of the Train is:', metrics.f1_score(y_train_raw, train_LGB_pre))
    print('The LightGBM Train confusion matrix result:\n', confusion_matrix_result)

    """ LightGBM testing results on the testing set """
    confusion_matrix_result = metrics.confusion_matrix(y_test, test_LGB_pre)
    print('The Lightgbm accuracy of the Test is:', metrics.accuracy_score(y_test, test_LGB_pre))
    print('The Lightgbm F1 of the Test is:', metrics.f1_score(y_test, test_LGB_pre))
    print('The LightGBM Test confusion matrix result:\n', confusion_matrix_result)
    y_test_predprob.append(test_LGB_proba[:, 1])
    plt.figure(figsize=(8, 6))
    sb.heatmap(confusion_matrix_result, annot=True, cmap='Blues', fmt='g')
    plt.xlabel('Predicted labels')
    plt.ylabel('True labels')
    plt.show()
    """ Return F1 score and prediction probability as the input of Weight """
    return metrics.f1_score(y_test, test_LGB_pre), test_LGB_proba,
```

```
metrics.f1_score(y_train_raw, train_LGB_pre), train_LGB_proba

def traintest_RF(X_train, y_train, X_test,y_test,X_train_raw, y_train_raw):
        """     Random Forest module    """
        # clf=RandomForestClassifier(n_estimators = 400, max_depth = 20, min_samples_split = 2,
min_samples_leaf = 1,max_features = 'auto',
        #                              n_jobs = 4,)
        """ can use    other hyperparameters """
        clf = RandomForestClassifier(n_estimators=140)
        clf.fit(X_train, y_train)
        test_RF_pre = clf.predict(X_test)
        test_RF_proba = clf.predict_proba(X_test)

        """ Random Forest training results on the original training set """
        train_RF_pre = clf.predict(X_train_raw)
        train_RF_proba = clf.predict_proba(X_train_raw)
        y_train_predprob.append(train_RF_proba[:,1])
        confusion_matrix_result = metrics.confusion_matrix(y_train_raw, train_RF_pre)
        print('The RF accuracy of the Train is:', metrics.accuracy_score(y_train_raw, train_RF_pre))
        print('The RF F1 of the Train is:', metrics.f1_score(y_train_raw, train_RF_pre))
        print('The RF Train confusion matrix result:\n', confusion_matrix_result)

        """ Random Forest testing results on the testing set """
        confusion_matrix_result = metrics.confusion_matrix(y_test, test_RF_pre)
        print('The RF accuracy of the Test is:', metrics.accuracy_score(y_test, test_RF_pre))
        print('The RF F1 of the Test is:', metrics.f1_score(y_test, test_RF_pre))
        print('The RF Test confusion matrix result:\n', confusion_matrix_result)
        y_test_predprob.append(test_RF_proba[:, 1])
        plt.figure(figsize=(8, 6))
        sb.heatmap(confusion_matrix_result, annot=True, cmap='Blues', fmt='g')
        plt.xlabel('Predicted labels')
        plt.ylabel('True labels')
        plt.show()
        """ Return F1 score and prediction probability as the input of Weight """
        return  metrics.f1_score(y_test,  test_RF_pre),  test_RF_proba,  metrics.f1_score(y_train_raw,
train_RF_pre), train_RF_proba

def PreDataset(filepath1):
        data1=np.loadtxt(filepath1,dtype=str,skiprows=1,delimiter=',')
        X, y = data1[:, 1:-1], data1[:, -1]
        """ 70% of training set """
        X_train, X_test, y_train, y_test = train_test_split(X, y, test_size=0.3, random_state=1)
        train = np.column_stack((X_train,y_train))
        np.savetxt('File_path_Train', train, fmt='%s', delimiter = ',')
```

```
	""" 30% of testing set """
	test = np.column_stack((X_test, y_test))
	np.savetxt('File_path_Test', test, fmt='%s', delimiter = ',')
	return X,y


def XGBoost_cv(X_train,y_train):
	""" Use grid search to adjust the hyperparameter in XGBoost """
	X_train = np.array(X_train)
	y_train = np.array(y_train)
	cv_params = {'n_estimators': [150,160,170,180,190]}
	""" Some optional hyperparameter """
	other_params = {"""'learning_rate': 0.3, 'n_estimators': 350, 'max_depth': 9, 
'min_child_weight': 1, 'seed': 0,
	                    'subsample': 0.7, 'colsample_bytree': 0.8, 'gamma': 0.1, 'reg_alpha': 3, 
'reg_lambda': 2,
	                    'scale_pos_weight': 8"""}
	model = xgb.XGBClassifier(**other_params)
	""" scoring:F1,accuracy,recall... """
	optimized_GBM = GridSearchCV(estimator=model, param_grid=cv_params, scoring='f1', 
cv=5, verbose=1, n_jobs=4)
	optimized_GBM.fit(X_train, y_train)
	evalute_result = optimized_GBM.cv_results_
	print('XGB results of each iteration:{0}'.format(evalute_result))
	print('XGB optimum value of parameter： {0}'.format(optimized_GBM.best_params_))
	print('XGB best model score:{0}'.format(optimized_GBM.best_score_))

def LightGBM_cv(X_train,y_train):
	""" Use grid search to adjust the hyperparameter in LightGBM """
	X_train = np.array(X_train)
	y_train = np.array(y_train)
	cv_params = {'n_estimators':[70,80,90,100,110]}
	""" Some optional hyperparameter """
	other_params = {"""'num_leaves': 35, 'learning_rate': 0.04, 'n_estimators': 500, 'max_depth': 
7, 'subsample': 0.01,
	                    'colsample_bytree': 0.5,
	                    'min_child_samples': 7, 'min_child_weight': 0.001"""}
	model = gbm.LGBMClassifier(**other_params)
	""" scoring:F1,accuracy,recall... """
	optimized_GBM = GridSearchCV(estimator=model, param_grid=cv_params, scoring='f1', 
cv=5, verbose=1, n_jobs=4)
	optimized_GBM.fit(X_train, y_train)
	evalute_result = optimized_GBM.cv_results_
	print('LGB results of each iteration:{0}'.format(evalute_result))
```

```
        print('LGB optimum value of parameter：{0}'.format(optimized_GBM.best_params_))
        print('LGB best model score:{0}'.format(optimized_GBM.best_score_))

def RF_cv(X_train,y_train):
        """ Use grid search to adjust the hyperparameter in Random Forest """
        X_train = np.array(X_train)
        y_train = np.array(y_train)
        cv_params = {'n_estimators':[100,110,120,130,140,150]}
        """ Some optional hyperparameter """
        other_params = {"""'n_estimators': 400, 'max_depth': 20, 'min_samples_split': 2,
'min_samples_leaf': 1,
                        'max_features': 'auto',
                        'n_jobs': 4, 'random_state': 10, 'min_weight_fraction_leaf': 0"""}
        model = RandomForestClassifier(**other_params)
        """ scoring:F1,accuracy,recall... """
        optimized_GBM = GridSearchCV(estimator=model, param_grid=cv_params, scoring='f1',
cv=5, verbose=1, n_jobs=4)
        optimized_GBM.fit(X_train, y_train)
        evalute_result = optimized_GBM.cv_results_
        print('RF results of each iteration:{0}'.format(evalute_result))
        print('RF optimum value of parameter：{0}'.format(optimized_GBM.best_params_))
        print('RF best model score:{0}'.format(optimized_GBM.best_score_))

def Weight_model(XGB_proba, XGB_F1, LGB_proba, LGB_F1, RF_proba, RF_F1, y):
      #Weight Model
      data_num = len(XGB_proba)
      weight_xgb = XGB_F1/(XGB_F1 + LGB_F1 + RF_F1)
      weight_lgb = LGB_F1/(XGB_F1 + LGB_F1 + RF_F1)
      weight_rf = RF_F1/(XGB_F1 + LGB_F1 + RF_F1)
      # Weighting the prediction probability of multiple models by F1 score
      Predict_Mix = []
      for row in range(0, data_num):
            tmp_list = []
            tmp_list.append(XGB_proba[row][0] * weight_xgb + LGB_proba[row][0] * weight_lgb +
RF_proba[row][0] * weight_rf)
            tmp_list.append(XGB_proba[row][1] * weight_xgb + LGB_proba[row][1] * weight_lgb +
RF_proba[row][1] * weight_rf)
            Predict_Mix.append(tmp_list)

      Predict_Mix = np.array(Predict_Mix)
      # Distinguish between the weight results of training or testing according to the number of
data
      if data_num < 300:
            # Use the testing result as input to the ROC-PR plot function
```

```
        y_test_predprob.append(Predict_Mix[:, 1])
    else:
        # Use the training result as input to the ROC-PR plot function
        y_train_predprob.append(Predict_Mix[:, 1])

    # Convert the comprehensive prediction probability to the prediction label by 0.5 threshold
    Predict_Mix = np.int64(Predict_Mix[:, 1]>0.5)
    print('The Weight model accuracy is:', metrics.accuracy_score(y, Predict_Mix))
    print('The Weight model F1 is:', metrics.f1_score(y, Predict_Mix))
    confusion_matrix_result = metrics.confusion_matrix(y, Predict_Mix)
    print('The MixModel confusion matrix result:\n', confusion_matrix_result)
    print(metrics.classification_report(y, Predict_Mix))
    plt.figure(figsize=(8, 6))
    sb.heatmap(confusion_matrix_result, annot=True, cmap='Blues', fmt='g')
    plt.xlabel('Predicted labels')
    plt.ylabel('True labels')
    plt.show()

def Weight_Stack_model(XGB_proba, XGB_F1, Stack_proba, Stack_F1, y):
      # Weight-Stack model
    data_num = len(XGB_proba)
    weight_xgb = XGB_F1/(XGB_F1    + Stack_F1)
    weight_sclf = Stack_F1/(tXGB_F1    + Stack_F1)
      # Weighting the prediction probability of Stack model and XGBoost by F1 score
    Predict_Mix = []
    for row in range(0, data_num):
        tmp_list = []
        tmp_list.append(XGB_proba[row][0] * weight_xgb + Stack_proba[row][0] * weight_sclf)
        tmp_list.append(XGB_proba[row][1] * weight_xgb + Stack_proba[row][1] * weight_sclf)
        Predict_Mix.append(tmp_list)

    Predict_Mix=np.array(Predict_Mix)
      # Weighting the prediction probability of multiple models by F1 score
    if data_num < 300:
        # Use the testing result as input to the ROC-PR plot function
        y_test_predprob.append(Predict_Mix[:, 1])
    else:
        # Use the training result as input to the ROC-PR plot function
        y_train_predprob.append(Predict_Mix[:, 1])

      # Convert the comprehensive prediction probability to the prediction label by 0.5 threshold
    Predict_Mix=np.int64(Predict_Mix[:,1]>0.5)

    print('The Weight-Stack accuracy is:', metrics.accuracy_score(y, Predict_Mix))
```

```
    print('The Weight-Stack F1 is:', metrics.f1_score(y, Predict_Mix))
    confusion_matrix_result = metrics.confusion_matrix(y,Predict_Mix)
    print('The Weight-Stack confusion matrix result:\n', confusion_matrix_result)
    print(metrics.classification_report(y, Predict_Mix))
    plt.figure(figsize=(8, 6))
    sb.heatmap(confusion_matrix_result, annot=True, cmap='Blues', fmt='g')
    plt.xlabel('Predicted labels')
    plt.ylabel('True labels')
    plt.show()

def Draw_ROC_PR(names, y_test_predprob, colors, y_test, dpin = 100):
    """
    ROC and PR curves of multiple machine learning models are output to one graph

    Args:
        names: list, Names of multiple models
        y_test_predprob: list, Probability predictors of multiple models
    """
    plt.figure(figsize=(20, 20), dpi=dpin)
    """ ROC """
    for (name, y_predprob, colorname) in zip(names, y_test_predprob, colors):
        fpr, tpr, thresholds = roc_curve(y_test, y_predprob, pos_label=1)
        plt.plot(fpr, tpr, lw=3, label='{} (AUC={:.3f})'.format(name, auc(fpr, tpr)),
color=colorname)
        plt.plot([0, 1], [0, 1], '--', lw=5, color='grey')
        plt.axis('square')
        plt.xlim([0, 1])
        plt.ylim([0, 1])
        plt.xlabel('False Positive Rate', fontsize=20)
        plt.ylabel('True Positive Rate', fontsize=20)
        plt.title('ROC Curve', fontsize=25)
        plt.legend(loc='lower right', fontsize=10)
    plt.grid()
    plt.show()

    """ PR """
    for (name, y_predprob, colorname) in zip(names, y_test_predprob, colors):
        precision, recall, thresholds = precision_recall_curve(y_test, y_predprob,pos_label=1)
        plt.plot(precision, recall, lw=3, label='{} (AUC={:.3f})'.format(name,
average_precision_score(y_test, y_predprob)), color=colorname)
        plt.plot([0, 1], [1, 0], '--', lw=5, color='grey')
        plt.axis('square')
        plt.xlim([0, 1])
        plt.ylim([0, 1])
```

```
            plt.xlabel('Recall', fontsize=20)
            plt.ylabel('Precision', fontsize=20)
            plt.title('Precision-Recall Curve', fontsize=25)
            plt.legend(loc='lower right', fontsize=10)
        plt.grid()
        plt.show()

def multi_models_calibration_curve(names, y_test_predprob, colors, y_test, dpin=100):
        # Drawing of calibration curves
        plt.figure(figsize=(20, 20), dpi=dpin)

        for (name, y_predprob, colorname) in zip(names, y_test_predprob, colors):

            y_means, proba_means = calibration_curve(y_test, y_predprob, strategy='quantile')
            plt.plot(proba_means, y_means, lw=3, label='{}'.format(name), color=colorname)
            plt.plot([0, 1], [0, 1], '--', lw=5, color='grey')
            plt.axis('square')
            plt.xlim([0, 1])
            plt.ylim([0, 1])
            plt.xlabel('Predicted probablity', fontsize=20)
            plt.ylabel('Target Variable', fontsize=20)
            plt.title('Calibration Curve', fontsize=25)
            plt.legend(loc='lower right', fontsize=10)
        plt.grid()
        plt.show()

if __name__ == '__main__':
        """ List of stored prediction probabilities of each model in the testing set """
        y_test_predprob = []
        """ List of stored prediction probabilities of each model in the training set """
        y_train_predprob = []
        """ Partition dataset """
        X,y = PreDataset('Dataset_filepath.csv')
        np.set_printoptions(suppress=True)
        """ File reading path of training set and testing set """
        trainFilePath = 'Train_file_path.csv'
        testFilePath = 'Test_file_path.csv'
        data = loadDataset(trainFilePath)

        """ Original Training Set """
        X_train_raw, y_train_raw = featureSet(data)
        X_test, y_test = loadTestData(testFilePath)

        """ Initialize the SHAP module """
```

```
shap.initjs()
X_train_raw = np.array(X_train_raw)
y_train_raw = np.array(y_train_raw)

""" SMOTEEN algorithm enhances and balances training set """
sm = SMOTEENN()
X_train, y_train = sm.fit_resample(X_train_raw, y_train_raw)
X_train = np.array(X_train)
X_test = np.array(X_test)
""" Cross validation tuning hyperparameter """
#XGBoost_cv(X_train, y_train)
#LightGBM_cv(X_train, y_train)
#RF_cv(X_train, y_train)
""" Get the training and testing performance of the benchmark model """
test_RF_F1, test_RF_proba, train_RF_F1, train_RF_proba = traintest_RF(X_train, y_train, X_test, y_test, X_train_raw, y_train_raw)
test_XGB_F1, test_XGB_proba, train_XGB_F1, train_XGB_proba = traintest_XGB(X_train, y_train, X_test, y_test, X_train_raw, y_train_raw)
test_LGB_F1, test_LGB_proba, train_LGB_F1, train_LGB_proba = traintest_LGB(X_train, y_train, X_test, y_test, X_train_raw, y_train_raw)

""" Logistic regression """
""" training """
LR = LogisticRegression()
LR.fit(X_train, y_train)
train_LR_pre = LR.predict(X_train_raw)
confusion_matrix_result = metrics.confusion_matrix(y_train_raw, train_LR_pre)
print('The LR Train confusion matrix result:\n', confusion_matrix_result)
train_LR_proba = LR.predict_proba(X_train_raw)
y_train_predprob.append(train_LR_proba[ :, 1])

""" testing """
test_LR_pre = LR.predict(X_test)
test_LR_proba = LR.predict_proba(X_test)
test_LR_proba = test_LR_proba[:, 1]
y_test_predprob.append(test_LR_proba)
confusion_matrix_result = metrics.confusion_matrix(y_test, test_LR_pre)
print('The LR confusion matrix result:\n', confusion_matrix_result)
plt.figure(figsize = (8, 6))
sb.heatmap(confusion_matrix_result, annot = True, cmap='Blues', fmt='g')
plt.xlabel('Predicted labels')
plt.ylabel('True labels')
plt.show()
```

```
""" Stacking Model """
lr = LogisticRegression()
RF = RandomForestClassifier(n_estimators = 140)
LGB = gbm.LGBMClassifier(n_estimators = 100)
XGB = xgb.XGBClassifier(n_estimators = 160, scale_pos_weight = 5)
""" 3 base learners and 1 meta learner """
sclf = StackingCVClassifier(classifiers=[RF, LGB, XGB], meta_classifier=lr, cv=5, use_probas=True)
""" training """
sclf.fit(X_train, y_train)
train_Stack_pre = sclf.predict(X_train_raw)
train_Stack_proba = sclf.predict_proba(X_train_raw)
y_train_predprob.append(train_Stack_proba[:, 1])
print('The Stack accuracy of the Train is:', metrics.accuracy_score(y_train_raw, train_Stack_pre))
print('The Stack F1 of the Train is:', metrics.f1_score(y_train_raw, train_Stack_pre))
train_Stack_F1 = metrics.f1_score(y_train_raw, train_Stack_pre)
confusion_matrix_result = metrics.confusion_matrix(y_train_raw, train_Stack_pre)
print('The STACK Train confusion matrix result:\n', confusion_matrix_result)
""" testing """
test_Stack_pre = sclf.predict(X_test)
test_Stack_proba = sclf.predict_proba(X_test)
y_test_predprob.append(test_Stack_proba[:, 1])

""" SHAP for Stack """
explainer = shap.KernelExplainer(sclf.predict_proba,X_test)
shap_values = explainer.shap_values(X_test)
""" feature_names : Feature names array in dataset """
""" Group level analysis chart of SHAP """
shap.summary_plot(shap_values[1], X_test, feature_names = ['Feature_1', 'Feature_2', 'Feature_3'])
shap.summary_plot(shap_values[1], X_test, plot_type = "bar",color = '#92C7BE',feature_names = ['Feature_1', 'Feature_2', 'Feature_3'])
""" Individual level analysis chart of SHAP """
Y_shap = np.array(y_test)
for number in range(0, 100):
    if (Y_shap[number] == 0.0):
        shap.force_plot(explainer.expected_value[1], shap_values[1][number, :], X_test[number, :], feature_names=['Feature_1','Feature_2','Feature_3'], matplotlib=True, show=False)
for number in range(50, 100):
    if (Y_shap[number] == 1.0):
        shap.force_plot(explainer.expected_value[1], shap_values[1][number, :], X_test[number, :], feature_names=['Feature_1','Feature_2','Feature_3'],
```

```
matplotlib=True,show=False)
                """ Save path """
                plt.savefig("Save_path" + str(number) + ".png")

    print('The Stack accuracy of the Test is:', metrics.accuracy_score(y_test, test_Stack_pre))
    print('The Stack F1 of the Test is:', metrics.f1_score(y_test, test_Stack_pre))
    test_Stack_F1 = metrics.f1_score(y_test, test_Stack_pre)
    confusion_matrix_result = metrics.confusion_matrix(y_test,test_Stack_pre)
    print('The Stack confusion matrix result:\n', confusion_matrix_result)
    plt.figure(figsize=(8, 6))
    sb.heatmap(confusion_matrix_result, annot=True, cmap='Blues', fmt='g')
    plt.xlabel('Predicted labels')
    plt.ylabel('True labels')
    plt.show()

    """ Weight Model train"""
    Weight_model(train_XGB_proba, train_XGB_F1, train_LGB_proba, train_LGB_F1,
train_RF_proba, train_RF_F1, y_train_raw)
    """ Weight-Stack Model train"""
    Weight_Stack_model(train_XGB_proba, train_XGB_F1, train_Stack_proba, train_Stack_F1,
y_train_raw)
    """ Draw ROC and PR curves of all models in training """
    names = ['Random Forest',
             'XGBoost',
             'LightGBM',
             'LR',
             'Stack',
             'Weight',
             'Weight-Stack'
             ]
    colors = ['crimson',
              'orange',
              'gold',
              'gray',
              'mediumseagreen',
              'steelblue',
              'mediumpurple'
              ]
    Draw_ROC_PR(names, y_train_predprob, colors, y_train_raw)
    """ Weight Model test"""
    Weight_model(test_XGB_proba, test_XGB_F1, test_LGB_proba, test_LGB_F1,
test_RF_proba,test_RF_F1, y_test)
    """ Weight-Stack Model test"""
    Weight_Stack_model(test_XGB_proba, test_XGB_F1,test_Stack_proba, test_Stack_F1,
```

```
y_test)
        """ Draw ROC and PR curves of all models in testing """
        Draw_ROC_PR(names, y_test_predprob, colors, y_test)
        """ Draw Calibration curves of all models in testing """
        multi_models_calibration_curve(names, y_test_predprob, colors, y_test)
        """ Draw Calibration curves of all models in training """
        multi_models_calibration_curve(names, y_train_predprob, colors, y_train_raw)
```
