# Supplementary material for: Machine-learning-based risk stratification for probability of dying in patients with basal ganglia hemorrhage
Source: Sci Rep. 2022 Dec 5;12:21035. doi: 10.1038/s41598-022-25527-1 (PMC9722697; doi:10.1038/s41598-022-25527-1)
Supplement: Supplementary file 3 — Supplementary Figure 1. [file 41598_2022_25527_MOESM3_ESM.docx]

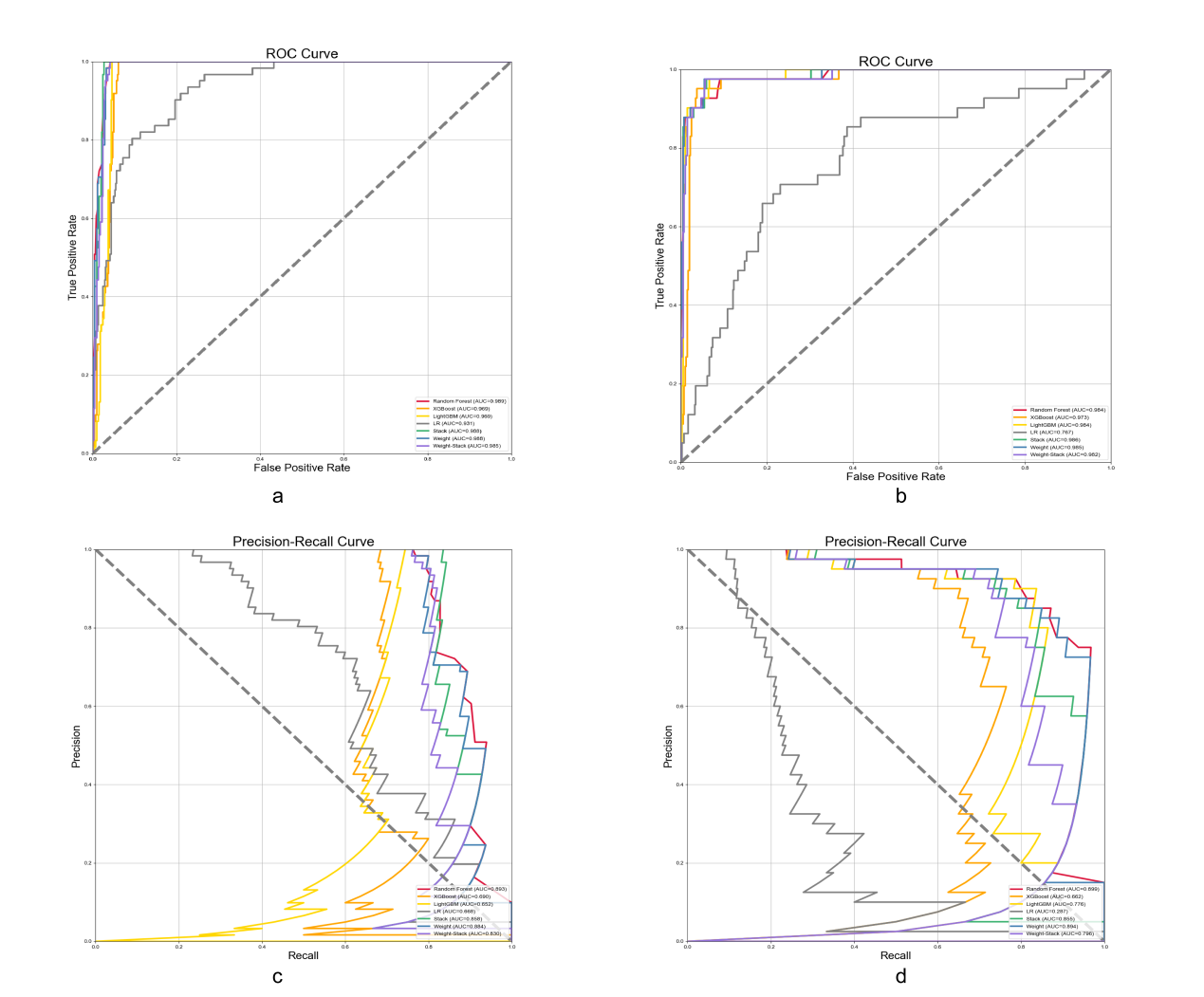


Supplementary Figure 1. Receiver operating characteristic curves and precision-recall curves on the training set for the conservative treatment group and the surgical treatment group. (a) Receiver operating characteristic curves for all models in the conservative treatment group. (b) Receiver operating characteristic curves for all models in the surgical treatment group. (c) Precision-recall curves for all models in the conservative treatment group. (d) Precision-recall curves for all models in the surgical treatment group.
