# Supplementary material for: Machine-learning-based risk stratification for probability of dying in patients with basal ganglia hemorrhage
Source: Sci Rep. 2022 Dec 5;12:21035. doi: 10.1038/s41598-022-25527-1 (PMC9722697; doi:10.1038/s41598-022-25527-1)
Supplement: Supplementary file 4 — Supplementary Figure 2. [file 41598_2022_25527_MOESM4_ESM.docx]

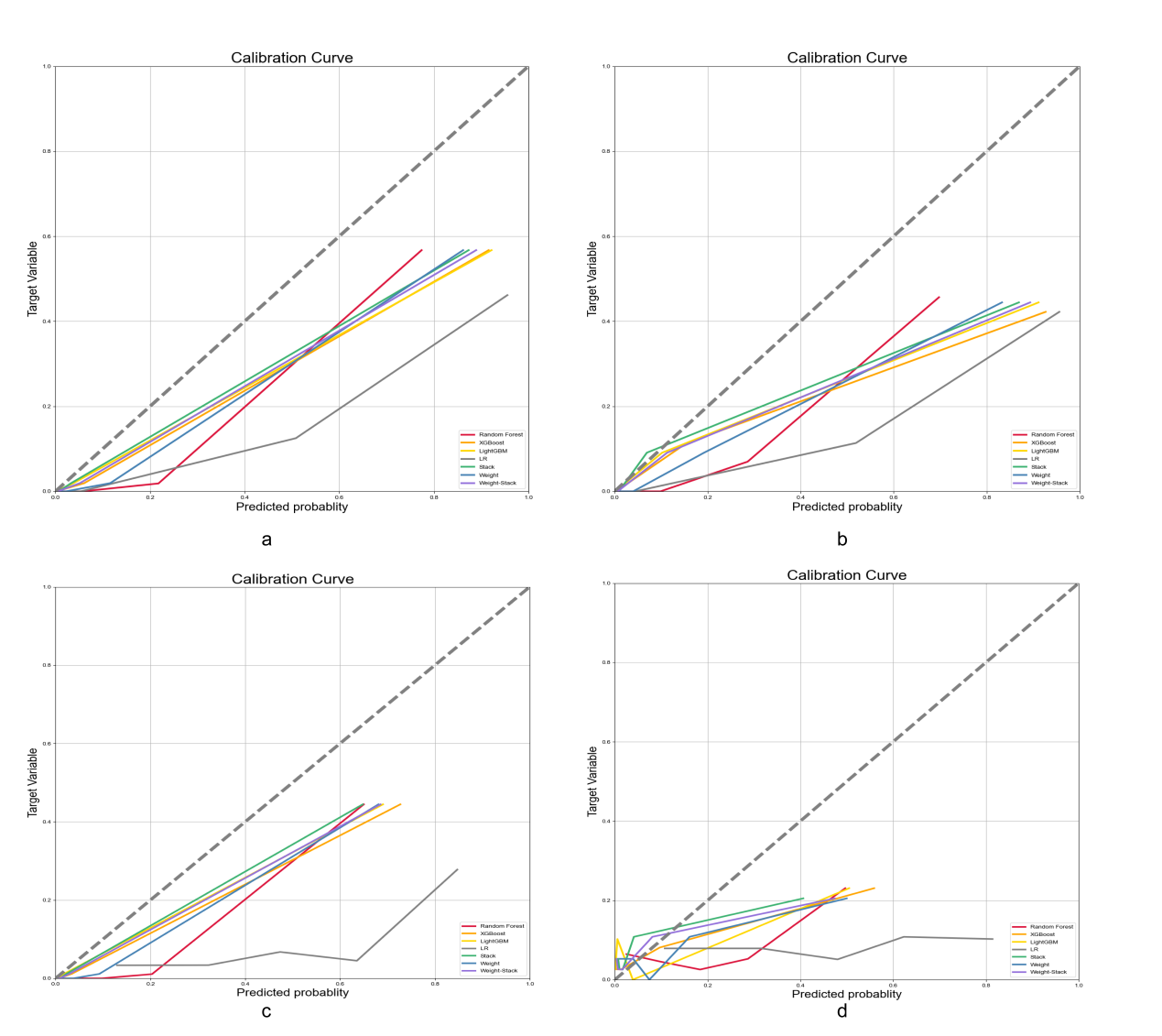


Supplementary Figure 2. Calibration curves for the conservative treatment group versus the surgical treatment group on the training set versus the testing set. (a) Calibration curves for all models in the conservative treatment group in the training set. (b) Calibration curves for all models in the surgical treatment group in the training set. (c) Calibration curves for all models in the conservative treatment group in the testing set. (d) Calibration curves for all models in the surgical treatment group in the testing set.
